# 

**Published:** 1880-08

**Authors:** 


### NEWS AGENTS

Will be supplied with HALL'S JOURNAL OF HEALTH by "The American News Company" and the "New York News Co." at reduced rates, commencing with January, 1880.

Post Masters, Publishers, Booksellers, and others, acting as Agents, can retain 50 cents out of each yearly subscription they send to us. No larger commission will be allowed in any case.

